# Using online wellness assessment to screen for risk of lowered work ability, burnout, depression and anxiety in occupational health: A cross-sectional study

**DOI:** 10.1177/20552076241274018

**Published:** 2024-09-09

**Authors:** Krista Kauppi, Katri Korpela, Patrik Borg, Eira Roos, Paulus Torkki

**Affiliations:** 1 Department of Public Health, Faculty of Medicine, 3835 University of Helsinki, Helsinki, Finland; 2 176478 Aava Medical Centre, Helsinki, Finland; 3 Research Programs Unit, Faculty of Medicine, 3835 University of Helsinki, Helsinki, Finland; 4 Department of Public Health, 3835 University of Helsinki, Helsinki, Finland

**Keywords:** Work Ability Score, Work Ability Index, well-being, domain, digital

## Abstract

**Objectives:**

An increasing prevalence of disability and sickness absences related to mental health highlights the need to find scalable measures to identify common occupational health challenges early on. This study (1) investigates how well current work ability measures capture psychosocial occupational health challenges, (2) examines how online wellness questionnaire data are linked to these challenges and (3) suggests a limited set of questions for screening employees.

**Methods:**

A total 709 employees filled out a wellness survey, the Work Ability Index, the Bergen Burnout Indicator and screening questions for generalized anxiety disorder and depression. The survey question clusters and previously identified domains of wellness were used to examine the correlations between the domains and occupational health indicators. Linear models and stepwise Akaike information criterion model reduction were used to identify questions that most explained variation in each challenge. The strongest questions were combined into a set, and recursive partitioning was used to form a screening tool for occupational health.

**Results:**

Despite over 80% of participants having good perceived work ability, we found a simultaneous anxiety risk in 22%, depression risk in 30%, some burnout symptoms in 7% and presenteeism in 36% of the participants. Correlations between several wellness domains and occupational health indicators were found. We identified eight questions that could be used to screen for a combined risk of lowered work ability, burnout, anxiety or depression.

**Conclusions:**

Our results demonstrate current measures not being sufficient to capture employees’ mental health and suggest a brief set of questions to identify employees at risk.

## Introduction

The changing work environment, with excessive working hours, non-standard forms of employment, remote work, teleworking and an ageing workforce, brings new challenges to the Nordic occupational health system.^1^ Issues related to mental health, with depression at the forefront, have become the main reason for work disability in Finland.^2^ Also, sickness absences due to anxiety disorders have risen steeply, especially among younger employees.^3^ This trend can lead to a higher disability burden and young people exiting the labour market early.^4^ Similar trends have been witnessed in other Nordic countries.^5^

Occupational health has a vital role in identifying employees at risk and targeting interventions to support and promote work ability to reduce adverse health, sickness absences, work disability and the costs associated with these.^6^ Work ability is a dynamic concept, which deals with the balance between working conditions and personal resources, and for this reason can vary depending on the job.^7,8^ Especially low organizational justice, high job demands and effort-reward imbalance have been found to increase the risk of work-related mental disorders and, thus, lower work ability.^9^ While the exact prevalence of these disorders is unclear, it has been estimated that the cost of work-related depression alone in European Union countries is over €600 billion every year and burnout, anxiety and presenteeism have been found to lower employee productivity and increase costs.^10–14^

Due to the increasing burden of these work-related mental disorders, more understanding is needed of how to prevent work ability from declining and turning into a work disability.^7^ Currently, the one-item Work Ability Score (WAS) and Work Ability Index (WAI) are often used in occupational health to assess employee work ability.^4,7^ However, due to the increasing burden of mental health challenges, it is vital to find additional methods to detect deteriorating work ability at an earlier stage.

Wellness is a positive state, more than an absence of disease, and it is considered to be dynamic and to include at least physical, social and mental domains.^15^ Studies have found that there is a clear link between wellness and many of the common mental health issues. Low levels of psychological wellness and poor health have been associated, for instance, with a risk of depression,^16^ generalized anxiety disorder (GAD),^17^ and burnout.^18^ All these consequently negatively affect employee work ability and productivity.^14,19,20^ Simultaneously, increasing the level of wellness has a chance to increase productivity, reduce absenteeism and presenteeism as well as lower healthcare costs.^21–25^

This study aims to (1) investigate how well current work ability measures (WAS and WAI) capture common psychosocial occupational health challenges (i.e. burnout, presenteeism and anxiety and depression risks), (2) investigate how different domains of wellness are linked to work ability measures and these common psychosocial challenges and (3) identify a set of questions that could be used in the occupational health setting to screen for risk of lowered work ability, burnout, anxiety and depression. The study offers a perspective on how using wellness data could support occupational health when identifying employees at risk.

## Methods

This cross-sectional study used WAI, Bergen Burnout Indicator (BBI-15) and Aisti wellness questionnaire data from Aava Medical Centre Ltd's (Aava's) Virta1 randomized controlled trial (RCT). The data were used from the trial's screening phase to investigate employee work ability and the prevalence of burnout, anxiety, depression and presenteeism in the Finnish employee population. We also used gender, age, body mass index and education data to describe the study population. To investigate how the wellness data are linked to the common occupational health indicators (WAI, WAS, BBI-15, GAD-2 Scale, The Whooley questions) and to identify questions to screen for risk of lowered work ability, questions from the existing Aisti well-being survey^26^ were clustered and placed under domains of wellness identified in previous research.^27–29^ These domains were Life satisfaction, Social network, Physical health, Mental health, Safety, Self-care and lifestyle habits, Meaningfulness, Work–life balance, Exercise, Nutrition, Community, Cognitive health and Sleep and recovery. Data from the Virta1 trial and linear regression were used to identify the questions that most explained each occupational health indicator. The strongest questions were combined into a set, and recursive partitioning was used to form a set of questions and question logic for screening purposes.

*Participants*. Three of Aava's occupational health client companies voluntarily participated in the trial from March 2020 to June 2021. Two of the companies were in the technology industry and had only white-collar office workers (*N* = 643) and one had both manual (*N* = 31) and office (*N* = 35) workers from the food industry. More detailed demographics of the participants can be found in Table 1. The trial has been registered at ClinicalTrials.gov (NCT04633876) and was approved by the Helsinki University Hospital District Ethics Committee on 4 September 2019 (HUS/2093/2019). All willing participants were invited to the screening phase but to be eligible for the study, they needed to be 18–65 years old, speak and understand Finnish or English, and have given written informed consent. As the Virta1 RCT trial was developed for employees with increased cardiometabolic risk and included a lifestyle intervention the exclusion criteria were (1) history of a major cardiovascular event during the past 6 months, (2) having type 1 or type 2 diabetes, (3) history of a malignant disease during the past 5 years, (4) use of lipid-lowering or obesity medications, (5) use of a cardiac pacemaker or history of atrial fibrillation, (6) pregnancy and (7) plan to travel more than one day a week during the intervention period.

**Table 1.** Demographics of the participants and prevalence of different conditions.

	Males		Females	
	N	Mean (SD)	N	Mean (SD)
Age	448	45.9 (8.7)	261	45.8 (8.7)
Body mass index	448	27.6 (12.2)	260	27.5 (15.9)
Variables				
Aisti questionnaire answers	443	69.3 (9.5)	257	67.6 (9.2)
Bergen Burnout Indicator	218	41.3 (11.5)	56	42.7 (13.0)
No burnout	133	33.8 (6.9)	31	32.9 (6.1)
Mild burnout	35	47.3 (1.8)	10	47.2 (1.5)
Mediocre burnout	40	55.0 (2.7)	9	55.9 (3.9)
Severe burnout	10	65.0 (3.6)	6	65.8 (1.3)
Work Ability Index	427	41.9 (4.6)	250	41.0 (4.9)
Poor work ability	6	24.1 (4.0)	5	23.3 (5.0)
Mediocre work ability	40	33.0 (2.5)	37	34.4 (2.1)
Good work ability	212	41.0 (1.9)	121	40.5 (2.0)
Excellent work ability	169	45.8 (1.5)	87	45.6 (1.4)
Work Ability Score	438	8.6 (1.1)	259	8.5 (1.3)
Whooley	445	0.5 (0.7)	259	0.9 (0.9)
Identified risk of depression	151	0.34 (0.47)	101	0.39 (0.49)
Generalized Anxiety Disorder Scale (GAD-2)	445	1.4 (1.5)	259	2.0 (1.6)
Identified risk of anxiety	102	0.23 (0.42)	89	0.35 (0.48)
	N	%	N	%
Education				
Non-vocational school	5	1%	10	4%
Vocational school	97	22%	65	25%
College	115	26%	62	24%
University	226	50%	123	47%
No information	5	1%	1	0%
Company				
1 (office workers, technology)	371	83%	201	77%
2 (office and manual workers, food)	33	7%	33	13%
3 (office workers, technology)	44	10%	27	10%
Presenteeism				
No presenteeism	253	58%	140	55%
5% productivity loss	128	29%	81	32%
10% productivity loss	37	8%	21	8%
15% productivity loss	13	3%	10	4%
20% productivity loss	5	1%	4	2%
26% productivity loss	1	0%	0	0%

SD: standard deviation.

*Data collection*. Demographic information, baseline measures of blood biomarkers, anthropometry and current work ability with the WAI were collected from all participants during the screening phase.^27^ Participants were also invited to fill in an Aisti well-being survey,^26^ which is an online tool developed by experts that includes 131 questions from nine different domains of wellness: a meaningful life, social relationships, mental health, work, financial situation, health promotion, sleep and recovery, activity and exercise and nutrition. The Aisti well-being score is calculated between 0 and 100 and most of the questions were rated on a continuous scale from 0 to 100. A higher score indicates a better response. The questions are mainly based on existing research literature and the expert group's clinical expertise. The assessment includes multiple scientifically validated instruments such as the GAD-2,^30^ two-item Whooley questions for screening depression risk,^31^ the Alcohol Use Disorders Identification Test^32^ and the Presenteeism Scale.^33^ The ratio of serum apolipoprotein B to apolipoprotein A1 concentrations was used to detect elevated cardiometabolic risk. The 45% of participants with the highest apolipoprotein ratio continued to an intervention phase and were asked to fill in BBI-15^34^ before the intervention. This paper does not focus on the intervention, which has been described in detail in a previous publication.^35^

*Investigated occupational health indicators*. The WAI was used to examine work ability. It examines work ability compared with lifetime best, work ability compared with mental and physical strains, number of current diseases diagnosed by a doctor, estimated work impairment due to these diseases, sick leave during the past 12 months, own prognosis of work ability 2 years from now, and mental resources.^36^ The total score varies between 7 and 49, where a score of 7–27 indicates poor work ability, 28–36 moderate, 37–43 good and a score higher than 43 an excellent work ability. Work ability was also assessed using solely the WAS, which is the question related to work ability compared with lifetime best. Work Ability Score is rated on a scale of 0–10, where points 0–5 signify poor work ability, 6–7 moderate, 8–9 good and 10 excellent work ability.^37^

A GAD-2 questionnaire was used to assess anxiety. The instrument included two questions: (1) ‘In the last 2 weeks, I have been bothered by feeling nervous, anxious or on edge’ and (2) ‘In the last 2 weeks, I have not been able to stop or control worrying’. Each question is scored on a scale of 0–3, totalling to 6 points. The recommended cutoff score is 3 points.^30^ To assess the risk of depression, the Whooley questions from the Primary Care Evaluation of Mental Disorders Patient Health Questionnaire (PHQ-4) were used. The used questions were: (1) ‘During the past month, have you often been bothered by feeling down, depressed or hopeless?’ and (2) ‘During the past month, have you been bothered by little interest or pleasure in doing things?’ Each question is rated on a scale of yes/no, where an answer ‘yes’ to one question indicates a positive screen. A visual analogue scale (0–100) was used for GAD-2 and the Whooley questions.^31^

Presenteeism was calculated using the Presenteeism Scale, which is based on the WAS, where a score of 8 results in 5% presenteeism, and the presenteeism grows by approximately 5% with each WAS point reduction.^33^ Work Ability Score scores 0–3 are not considered and scores 9–10 indicate 0% productivity loss. The BBI-15^34^ was used to assess burnout among participants. The instrument measures three dimensions of burnout: exhaustion, cynicism and professional efficacy using five questions for each dimension. The questions are evaluated on a 6-point Likert scale, where the options range from ‘completely disagree’ to ‘strongly agree’. The total score varies between 15 and 90, where a score of 15–44 signifies no burnout, 45–50 mild burnout, 51–60 moderate burnout and a score higher than 60 severe burnout.^34^ The instrument has been validated in large datasets and shown to be reliable in research and occupational health contexts.^38^

*Aisti survey question clusters*. A recent study analyzed the structure of the Aisti well-being survey using pairwise Spearman correlations between the questions, calculating distances as 1-correlation and using hierarchical clustering to group the questions at four levels into thematic categories.^27^ The questions inside the categories were used to name each category. The finest level of clustering included 25 question categories. Previous studies have identified, using an expert panel and Finnish laypeople, 13 different wellness domains.^28,29^ These were used to interpret the 25 question clusters to link the peer-reviewed results with the Aisti well-being assessment. Three of the authors independently reviewed the question clusters and assigned the most appropriate wellness domain to each of the clusters (Supplement 1). The fourth author acted as an arbiter of four question groups where consensus was not initially reached.

*Data analysis and synthesis*. The work ability results and the prevalence of different psychosocial occupational health challenges (burnout, presenteeism and risks of anxiety and depression) were compared using percentages. Correlations between the wellness domains and the investigated occupational health indicators were examined using a heat map (function heatmap.2 in R). As the original WAI, WAS and presenteeism data were skewed, the raw scores were transformed by squaring. Linear models were created for the 25 survey question clusters and, based on the Akaike information criterion (AIC), the question that most explained the variation was chosen from each question cluster. These were combined into a single multiple linear regression model that included only complete cases for each investigated indicator. To investigate in more detail how well the currently used work ability measure (WAS) of occupational health works in identifying burnout, anxiety and depression risk, the one-item work ability question was also added to the model for these three indicators. The model was reduced using stepwise AIC-based model reduction to select a set of non-collinear questions that best explained the investigated indicator. To find the questions that increased the adjusted R^2^ the most, the questions were combined one by one into a multiple linear regression model.

Of the six indicators, the questions that most explained the variation were combined into a set. If the next question increased explanatory power by less than 2%, the question was not included in the set. Recursive partitioning (function rpart in R) was used to form a regression tree by using the question set and categorizing the employees to either have a risk of any of the investigated outcomes or not to have a risk. An employee was coded to have a risk if (a) they had mediocre or severe burnout (BBI-15 > 50), (b) their self-rated work ability was less than ‘good’ (WAS < 8), (c) they had a risk of anxiety (GAD-2 ≥ 3) or (d) they had a risk of depression (Whooley points ≥ 1). The minimum number of observations in each node was set to 25 and the complexity parameter to 0.005.

## Results

A total of 713 participants were screened, of which 319 were randomized to individual lifestyle coaching (*N* = 53), group lifestyle coaching (*N* = 107) and control groups for individual lifestyle coaching (*N* = 53) and group lifestyle coaching (*N* = 106). This study focuses on the baseline measures and does not investigate the effect of the intervention. The majority of the screened participants were male (*N* = 448), with 261 females and four participants that specified their gender as ‘other’ or did not specify gender. The mean age was 45.9 years (standard deviation 8.7 years). All investigated indicators had missing values, with BBI-15 results missing the most (*N* = 45). Five of the participants were missing data on anxiety and depression risk, 13 were missing Aisti questionnaire results, 36 were missing a fully completed WAI and 16 were missing data on the WAS and presenteeism. More detailed demographics can be found in Table 1.

### Current work ability measures and prevalence of burnout, presenteeism and risk of depression and anxiety in a Finnish working-age population sample

Most of the participants had good work ability (89% of men, 83% of women), with poor work ability found in only 1% of men and 2% of women. However, participants with good work ability simultaneously experienced burnout symptoms in approximately 7% of cases (Table 2). Anxiety and depression risk were found in approximately 22% and 30% of participants with good work ability. When examined combined, approximately 42% of participants with ‘good’ or ‘excellent’ work ability simultaneously had anxiety or depression risk or experienced mediocre or severe burnout. Furthermore, some level of productivity loss was found in 36% of participants with good work ability.

**Table 2.** Participants with good work ability but simultaneous burnout or risk of anxiety or depression.

Work Ability Score						
Value	Total participants	Burnout	Anxiety risk	Depression risk	Burnout, anxiety or depression risk	Productivity loss (≥5%)
Excellent (10)	128	10 (7.8%)	19 (14.8%)	25 (19.5%)	41 (32.0%)	
Good (9)	265	12 (4.5%)	54 (20.4%)	74 (27.9%)	103 (38.9%)	
Good (8)	210	19 (9.0%)	64 (30.5%)	83 (39.5%)	114 (54.3%)	
Total	603	41 (6.8%)	137 (22.7%)	182 (30.2%)	258 (42.8%)	
Work Ability Index						
Work ability	Total participants	Burnout	Anxiety risk	Depression risk	Burnout, anxiety or depression risk	Productivity loss (≥5%)
Excellent (44–49)	256	8 (3.1%)	31 (12.1%)	46 (18.0%)	68 (26.6%)	37 (14.5%)
Good (37–43)	334	33 (9.9%)	97 (29.0%)	129 (38.6%)	180 (53.9%)	173 (51.8%)
Total	590	41 (6.9%)	128 (21.7%)	175 (29.7%)	248 (42.0%)	210 (35.6%)

### Links between domains of wellness and common occupational health indicators

**Figure 1.**
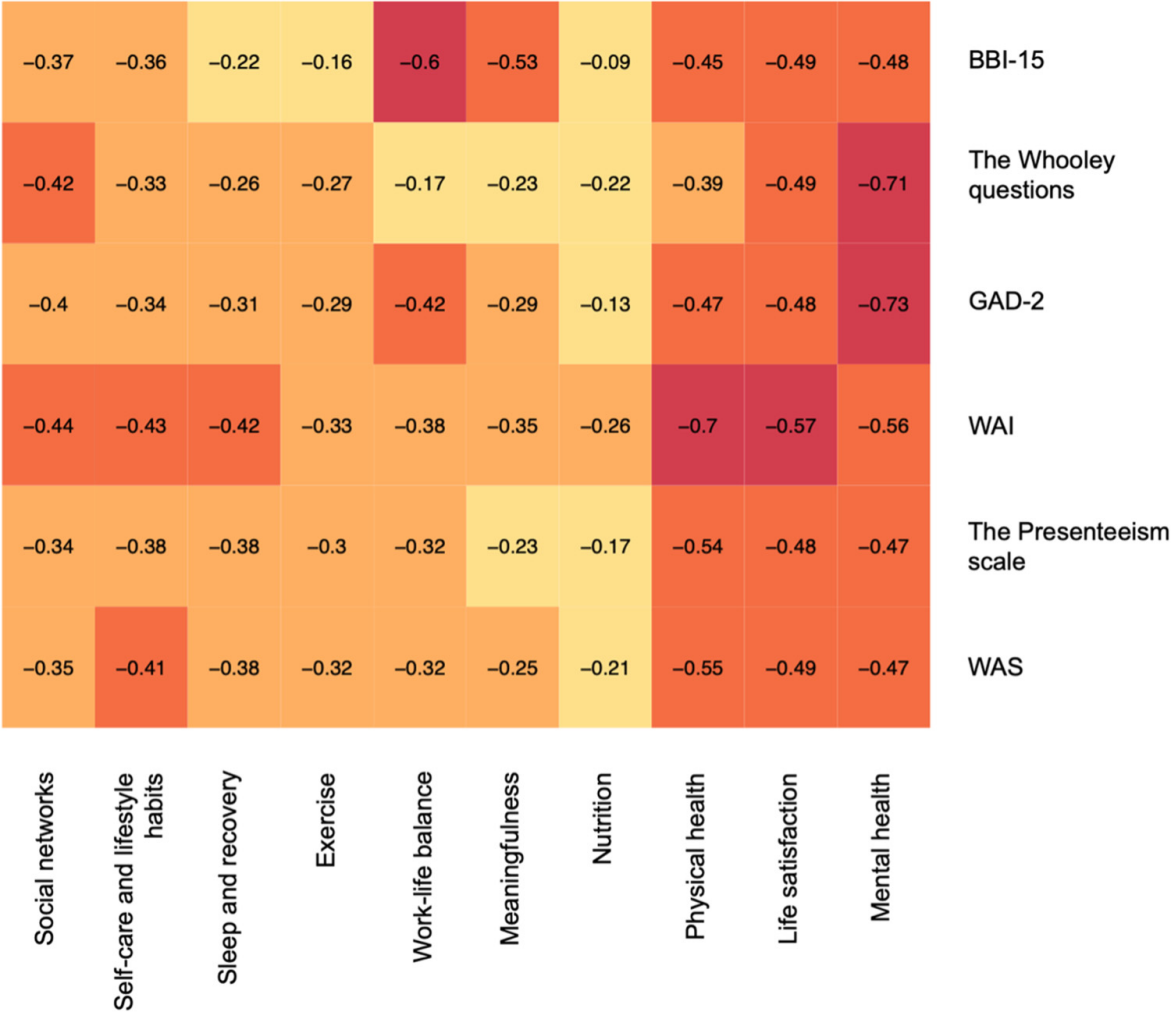
Correlations between different wellness domains and occupational health indicators. BBI-15: Bergen Burnout Indicator; GAD-2: Generalized Anxiety Disorder Scale; WAI: Work Ability Index; WAS: Work Ability Score. Darker colours signify a stronger correlation.

Each of the investigated occupational health indicators had correlations with many different wellness domains (Figure 1). Three of the domains – ‘Mental health’, ‘Life satisfaction’ and ‘Physical health’ – had stronger relationships with all indicators compared with the other domains. On average, each occupational health indicator had at least a weak relationship with eight different domains of wellness. The strongest correlations could be found between ‘Mental health’ questions and anxiety and depression risk. The burnout indicator had a moderate correlation with the domains ‘Meaningfulness’ and ‘Work-life balance’. The WAI had moderate relationships with the domains ‘Mental health’, ‘Life satisfaction’ and ‘Physical health’, the strongest being ‘Physical health’. The Presenteeism Scale and WAS are based on the same measure and have similar correlations, with the strongest being with the domain ‘Physical health’.

### A limited set of questions for screening employees in occupational health

Ten questions explained 53% of the variation in the BBI-15 (Supplement 2). Four of the questions – ‘We have a good community at work’, ‘My work demands more from me than I can give’, ‘It does not take me long to recover from a stressful event’ and ‘I would rate my fatigue as’ – explained 50% of the variation (Supplement 3). The commonly used work ability question (WAS) did not appear in the model. Changes in WAI scores were also explained up to 49% by nine questions, but three questions had an explanatory power of 44%. These were ‘Do you have a long-term disease and does it inhibit your life?’, ‘I’m satisfied with my ability to cope with daily activities’ and ‘In the past 2 weeks I have experienced a feeling of nervousness, anxiety or tension’. For the one-question work ability screen (WAS), the model identified 12 wellness questions that explained 32% of the changes in work ability. Most of this was explained with the questions ‘I’m satisfied with my ability to cope with daily activities’ and ‘In the past 2 weeks I have experienced a feeling of nervousness, anxiety or tension’ (25%).

In the two-item anxiety screen GAD-2, 13 questions explained 53% of the variation in the anxiety points. The questions ‘I feel stressed, tense, restless, nervous, anxious or have trouble sleeping’ and ‘I feel inner peace’ explained 49% of this variation. The WAS did appear in the model but had the lowest explanatory power. The model found eight questions that explained approximately 32% of the variation in depression risk. None of the eight questions were WAS. The questions ‘I feel happy and joyful’, ‘I have enough energy for daily activities’ and ‘In the past 2 weeks I have experienced a feeling of nervousness, anxiety or tension’ were able to explain approximately 29% of this variation. For presenteeism, the model identified nine questions that explained 29% of the variation in the presenteeism variable. The question ‘I’m satisfied with my ability to cope with daily activities’ explained 21% of this variation and with the questions ‘Overall I am happy with my job’ and ‘In the past 2 weeks I have experienced a feeling of nervousness, anxiety or tension’ the adjusted R^2^ rose to 25%. The questions that explained most of the variation for each indicator are listed in Table 3.

**Table 3.** Strongest questions for explaining variation in the Bergen Burnout Indicator (BBI-15), Work Ability Index (WAI), Work Ability Score (WAS), Generalized Anxiety Disorder Scale (GAD-2), Whooley and the presenteeism measures.

#	Indicator (positive/negative association)	Question
1	BBI-15 (−)	We have a good community at work
2	BBI-15 (−)	My work demands more from me than I can give
3	BBI-15 (−)	It does not take me long to recover from a stressful event
4	BBI-15 (−)	I would rate my level of fatigue as
5	WAI (+)	Do you have a long-term disease and does it inhibit your life?
6	WAI (+), WAS (+), Presenteeism Scale (−)	I’m satisfied with my ability to cope with daily activities
7	WAI (+), WAS (+), Whooley (−), Presenteeism Scale (−)	In the past 2 weeks I have experienced a feeling of nervousness, anxiety or tension
8	GAD-2 (−)	I feel stressed, tense, restless, nervous, anxious or have trouble sleeping
9	GAD-2 (−)	I feel inner peace
10	Whooley (−)	I feel happy and joyful
11	Whooley (−)	I have enough energy for daily activities
12	Presenteeism Scale (−)	Overall I am happy with my job

More details on p-values, estimates and standard errors can be found in Supplement 2.

**Figure 2.**
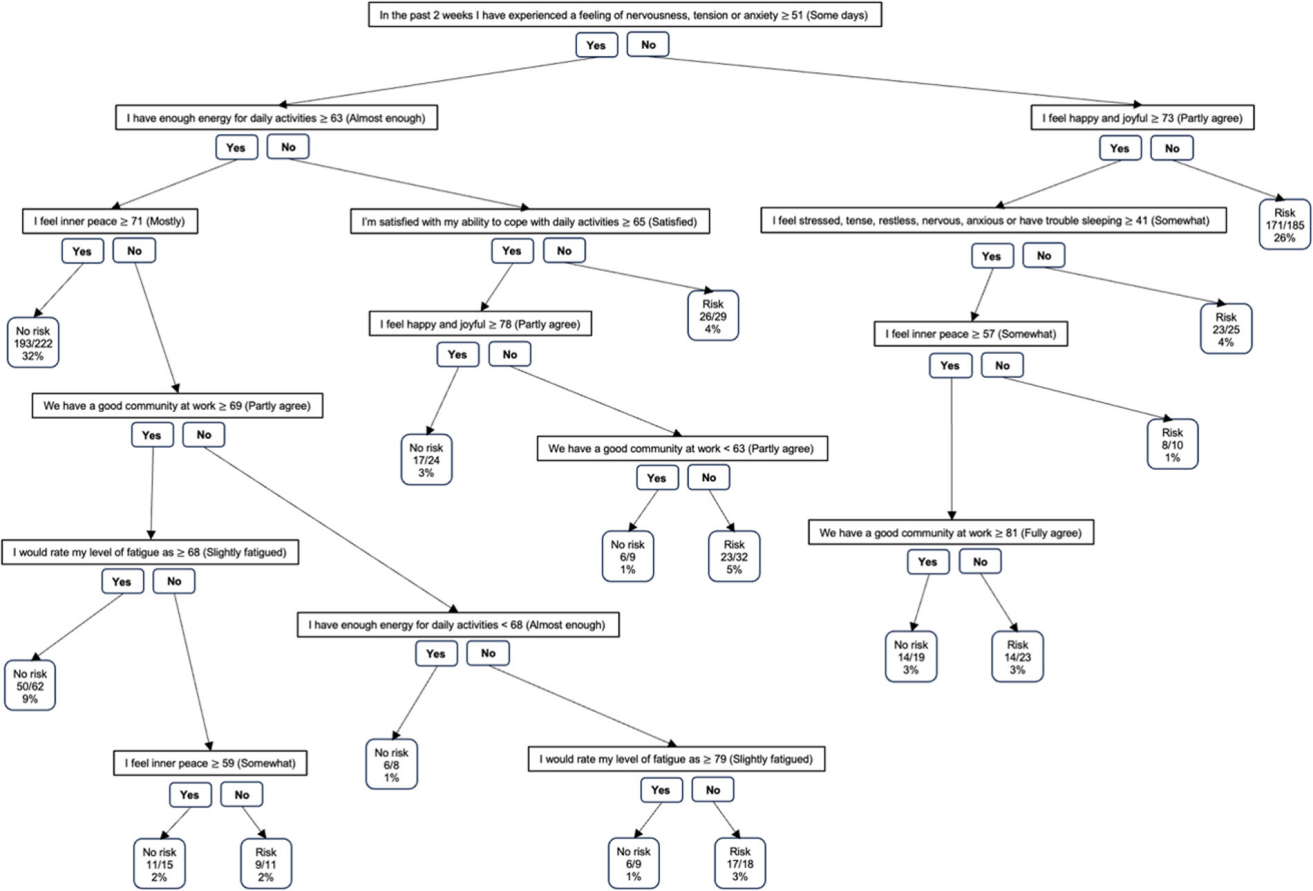
Set of eight questions and question logic for screening the risk of lowered work ability, burnout, depression and anxiety. Classification rates indicating the number of right classifications compared with the total number of observations together with the percentage of observations are presented.

The regression tree revealed that eight of the questions formed a logic for screening if there is a risk of either lowered work ability, burnout, anxiety or depression (Figure 2). These were ‘*We have a good community at work*’, ‘*I would rate my level of fatigue as*’, ‘*I’m satisfied with my ability to cope with daily activities*’, ‘*In the past 2 weeks, I have experienced a feeling of nervousness, anxiety or tension*’, ‘*I feel stressed, tense, restless, nervous, anxious or have trouble sleeping*’, ‘*I feel inner peace*’, ‘*I feel happy and joyful*’ and ‘*I have enough energy for daily activities*’. The maximum number of questions in each path was six and the model performed well, with an accuracy of 0.85 and Cohen's kappa of 0.7. The sensitivity of the model was 0.88 and the specificity was 0.82. The question points are presented on a continuous scale of 0–100, where values closer to 100 indicate better wellness. Supplement 4 presents the scales for each question.

## Discussion

Increases in mental health challenges have increased the need to find new methods that earlier identify employees at risk of lowered work ability. Hence, we wanted to understand how well current work ability measures capture different psychosocial occupational health challenges and examine how wellness data could be used for screening purposes. Despite over 80% of participants having good work ability, we found that approximately 42% of participants simultaneously had a risk of anxiety or depression or experienced mediocre or severe burnout. We also found that each of the investigated occupational health indicators correlated with many different wellness domains and were able to form a set of eight wellness questions that could be used to screen if there is a risk of any of the investigated outcomes (lowered work ability, anxiety, depression or burnout).

This study compared participants’ work ability evaluations with the prevalence of different psychosocial occupational health challenges. For the interpretation of results, it is important to note that compared with previous findings, the prevalence of burnout in our sample was higher both in men and women than in previous Finnish studies, where severe burnout was found in approximately 2%, and mild or mediocre burnout in 21–25%, of men and women.^39^ Possible reasons for this include the use of different screening instruments and our data being collected during the COVID-19 pandemic. For instance, our findings on the prevalence of GAD and depression risks resemble previous findings from the time of the COVID-19 pandemic, when the former was found in approximately 20%, and depression risk based on PHQ-4 in 37.7%, of respondents.^40^ However, some researchers have commented on the probable overestimation of anxiety and depression risks during the pandemic.^41^ It is good to remember that both GAD-2 and the Whooley questions are used as screening tools, where the cut-off point should prompt completion of the full instruments and a clinical interview. Furthermore, the Whooley questions have high sensitivity (0.95) but only modest specificity (0.65), whereas the sensitivity and specificity for GAD-2 are 0.86 and 0.83, respectively.^31,42^ This study found presenteeism in 43% of respondents, which is close to the figures for managers and engineers, as well as previous findings from the food industry, where presenteeism was 48%.^33^

Our findings on the level of work ability are close to previous Finnish studies, in which employed women in the age group 30–64 years had on average 8.7 points for self-perceived work ability and men 8.6, while the WAI points were 39.7 for women and 40.3 for men.^43^ Hence, our study population seems to represent previous studies quite well. However, based on our findings it seems that the WAS and WAI might not be sufficient measures to use in occupational health settings as many of the respondents suffered from burnout or had anxiety or depression risk despite considering their work ability to be good. The WAS also neither appeared in the models nor had strong explanatory power when identifying the most important questions for burnout, anxiety and depression. Previous research has provided similar indications about the disparity between estimated work ability and the prevalence of mental health challenges.^44^

When looking at the links between the different domains of wellness and occupational health indicators, we noticed that, on average, questions from approximately 8 out of 10 (83%) domains had at least a weak correlation with the investigated occupational health indicators and consequently play a role in employee work ability. In particular, the domains ‘Mental health’, ‘Life satisfaction’ and ‘Physical health’ seemed to be meaningful in all investigated indicators. The WAI had the strongest correlation with ‘Physical health’ questions, while the correlation with ‘Mental health’ was smaller. This seems to be in line with our results on work ability and the simultaneous presence of psychosocial occupational health challenges. Considering that there is no widely accepted agreement on the domains of wellness^45^ and that previous studies have attempted to harmonize the concept,^28,29^ these findings bring valuable insight into the most important domains of wellness from an occupational health perspective. These findings also highlight the need to examine employees from a broader perspective, including factors such as meaningfulness, self-care and lifestyle habits and social networks, instead of focusing mainly on health-related factors to support employees’ work ability.

When examining what kind of questions are behind these wellness domains, we were able to identify 2–4 most important questions for each occupational health indicator. The questions we identified for burnout are in line with previous findings, as workload, exhaustion and work community have been found to have an important role in burnout.^18^ Furthermore, our findings on the link between burnout and the ability to recover from stressful events match some previous findings.^46^ The questions that most explained the changes in the GAD-2 score (‘*I feel stressed, tense, restless, nervous, anxious or have trouble sleeping*’, ‘*I feel inner peace*’) are also rational as the question about feeling stressed is very similar to the question in GAD-2 and a lack of inner peace is the source of anxiety. However, it is noteworthy that these two very anxiety-related questions explain only approximately 50% of the variation and it would be interesting to further investigate where the other 50% comes from. The identified questions linked to depression risk dealt with happiness, energy and feelings of stress and anxiety, which are all linked to common symptoms of depression.^16^

The questions most explaining variation in the work ability measures were only slightly different for the WAI and WAS. The questions related to functioning as well as nervousness, tension and anxiety were found in both, indicating that both mental and physical health have an important role in work ability. As the WAI investigates long-term disease in more detail, it is not surprising that a question related to that appeared even though the same question was not found in the WAS analysis. However, what is noteworthy is that happiness with a job seemed to have some explanatory power in both instruments, which resembles previous findings on the importance of happiness at work.^47^ As the used presenteeism measure was based on the WAS, it makes sense that the questions explaining most of the variation in presenteeism are like the ones identified with the WAS.

Current occupational health screenings still rely largely on traditional health indicators (weight, blood pressure and blood biomarkers), work ability measures (WAI, WAS) and clinical interviews, which are often conducted for certain age groups (for instance all employees aged 40 years). These have worked fine in the past. However, society has changed vastly due to changes such as excessive working hours, non-standard forms of employment, remote work, teleworking and an ageing workforce.^1^ This study showed that WAI correlated best with employees’ physical health and while over 80% of respondents reported good work ability, there was a high prevalence of mental health challenges. As the mental health-related disability keeps on rising in Finland,^48^ the occupational health is still struggling to identify employees at risk at an early stage. Hence, we need to find new questions that capture a broader spectrum of employee wellness and potentially help in identifying mental health challenges earlier. This study presented eight questions, namely ‘*We have a good community at work*’, ‘*I would rate my level of fatigue as*’, ‘*I’m satisfied with my ability to cope with daily activities*’, ‘*In the past 2 weeks, I have experienced a feeling of nervousness, anxiety or tension*’, ‘*I feel stressed, tense, restless, nervous, anxious or have trouble sleeping*’, ‘*I feel inner peace*’, ‘*I feel happy and joyful*’ and ‘*I have enough energy for daily activities*’ that should be validated in a larger dataset. These questions cover domains of wellness from meaningfulness to physical and mental health and life satisfaction, offering a quick but versatile view of employee health.

Without new scalable screening methods, we continue to struggle with the high cost and disability burden that the psychosocial occupational health challenges carry. For instance, employees who rated their work ability good had 12–14 sickness absence days a year, while those who rated it mediocre had 22–23 and the ones who considered it poor, 44–75.^4^ Often these occupational health challenges can be seen as increases in presenteeism, which has been estimated to have cost around €7 billion in Finland in 2017 (€3262 per employee) through lowered productivity.^12^ By preventing severe burnout, cost savings could be up to €18,200 per employee, as it has been found to cause up to 52 excess sick days over 2 years.^11^ The total direct costs between depressed and non-depressed employees are 158% higher in adults, and indirect costs are 128% higher, while employees with an anxiety disorder had approximately 13 days more absences compared with a control group.^13,14^ Hence, the motivation to find and prevent these challenges early on is very high, and scalable online tools could be beneficial in identifying employees at risk.

### Strengths and limitations

We identified some limitations that could have affected the study. The Virta1 RCT started right when the COVID-19 pandemic came to Finland. Hence, this will likely have affected the prevalence of some psychosocial occupational health challenges. Furthermore, most of our study population consisted of male white-collar workers from the technology industry. Hence, results would likely differ in a more balanced sample. Furthermore, as the sample population was very skewed, we were not able to perform analyses based on gender or the type of work or industry. Hence, it may not be possible to generalize the results to other populations such as the unemployed. It is also important to note that BBI-15 was done for participants with increased cardiometabolic risk, which also affects the generalizability of the results.

Due to the design of the Virta1 trial, the sample burnout population was relatively small. However, from a statistical testing point of view, this population was considered to be sufficient. Furthermore, for certain occupational health indicators, the explanatory power of the questions was quite small (∼25%). From a methodological point of view, it would have been beneficial to combine multiple different instruments such that there would have been questions for all the domains identified in previous research. We also note that the eight-question screening tool is based on cross-sectional data and was not validated in this paper. Hence, a larger longitudinal study would be needed to examine the causal relationships and validate the tool. However, simultaneously we consider as strengths the use of the chosen survey as it consisted of 131 questions from various domains of wellness. The study also investigates multiple different occupational health indicators and sheds much-needed light on the possible wellness questions that could help in the clinical setting.

### Implications for occupational health practitioners

The focus of occupational health services is to support and enhance work ability and prevent work disability. However, during recent years, sickness absences due to mental disorders have increased in Nordic countries, especially in Finland,^3^ implying that current means to prevent mental disorders among the working-age population are not sufficient. This pressures occupational health services and client organizations to find new ways to earlier identify employees who are at risk of lowered work ability. Currently, the WAI and WAS are used in employee surveys to find those employees who need further examinations in the occupational healthcare system.^7,39^ This study shows that despite employees rating their work ability ‘good’, over 40% of them had symptoms of burnout or a risk of anxiety or depression. Thus, the WAS and WAI do not seem to be sufficient measures to identify those employees who require occupational health intervention.

It is known that work ability is the sum of different factors, of which health is only one.^49^ In our study, many domains of wellness were linked to the common occupational health indicators, signalling the need to examine employees’ lives from a broader angle. We demonstrate how an online wellness assessment and a very brief set of questions could be used to identify those employees who need occupational health system involvement. In practice, occupational health practitioners could invite employees, who have been classified as having a risk for a health examination. In contrast, other employees could receive a message indicating they can contact occupational health if necessary. However, the set should be validated in a different data set and in actual clinical use to examine the accuracy of the question set in practice and whether practitioners find it useful.

## Conclusions

We identified the need to incorporate alternative measures, in addition to the WAS and WAI, that examine employee wellness from a broader angle to identify employees at risk of burnout, depression and anxiety. Furthermore, the study presents a brief set of questions and a question logic that could be validated further and used to create a scalable online screening tool for occupational health.

## Supplemental Material

sj-docx-1-dhj-10.1177_20552076241274018 - Supplemental material for Using online wellness assessment to screen for risk of lowered work ability, burnout, depression and anxiety in occupational health: A cross-sectional study Supplemental material, sj-docx-1-dhj-10.1177_20552076241274018 for Using online wellness assessment to screen for risk of lowered work ability, burnout, depression and anxiety in occupational health: A cross-sectional study by Krista Kauppi, Katri Korpela, Patrik Borg, Eira Roos and Paulus Torkki in DIGITAL HEALTH

sj-xlsx-2-dhj-10.1177_20552076241274018 - Supplemental material for Using online wellness assessment to screen for risk of lowered work ability, burnout, depression and anxiety in occupational health: A cross-sectional study Supplemental material, sj-xlsx-2-dhj-10.1177_20552076241274018 for Using online wellness assessment to screen for risk of lowered work ability, burnout, depression and anxiety in occupational health: A cross-sectional study by Krista Kauppi, Katri Korpela, Patrik Borg, Eira Roos and Paulus Torkki in DIGITAL HEALTH

sj-docx-3-dhj-10.1177_20552076241274018 - Supplemental material for Using online wellness assessment to screen for risk of lowered work ability, burnout, depression and anxiety in occupational health: A cross-sectional study Supplemental material, sj-docx-3-dhj-10.1177_20552076241274018 for Using online wellness assessment to screen for risk of lowered work ability, burnout, depression and anxiety in occupational health: A cross-sectional study by Krista Kauppi, Katri Korpela, Patrik Borg, Eira Roos and Paulus Torkki in DIGITAL HEALTH

sj-docx-4-dhj-10.1177_20552076241274018 - Supplemental material for Using online wellness assessment to screen for risk of lowered work ability, burnout, depression and anxiety in occupational health: A cross-sectional study Supplemental material, sj-docx-4-dhj-10.1177_20552076241274018 for Using online wellness assessment to screen for risk of lowered work ability, burnout, depression and anxiety in occupational health: A cross-sectional study by Krista Kauppi, Katri Korpela, Patrik Borg, Eira Roos and Paulus Torkki in DIGITAL HEALTH
