# Supplementary material for: Using online wellness assessment to screen for risk of lowered work ability, burnout, depression and anxiety in occupational health: A cross-sectional study
Source: Digit Health. 2024 Sep 9;10:20552076241274018. doi: 10.1177/20552076241274018 (PMC11384527; doi:10.1177/20552076241274018)
Supplement: sj-docx-1-dhj-10.1177_20552076241274018 - Supplemental material for Using online wellness assessment to screen for risk of lowered work ability, burnout, depression and anxiety in occupational health: A cross-sectional study [file sj-docx-1-dhj-10.1177_20552076241274018.docx]

**Supplement 1**

The finest level of Aisti survey clusters and assigned wellness domains.

| Aisti questionnaire finest level of clustering | wellness domain |
| --- | --- |
| Happiness | Life satisfaction |
| Relationships | Social network |
| Energy for daily life | Physical health |
| Resilience | Mental health |
| Mental well-being challenges | Mental health |
| Time for oneself | Self-care and lifestyle habits |
| Work satisfaction | Meaningfulness |
| Workload | Work-life balance |
| Financial prudence | Not applicable |
| Satisfaction with salary | Life satisfaction |
| Control of emotions | Social networks |
| Flexible mindset | Self-care and lifestyle habits |
| Physical activity | Exercise |
| Diet | Nutrition |
| Alcohol and tobacco use | Self-care and lifestyle habits |
| Eating behaviour | Self-care and lifestyle habits |
| Daily activity | Exercise |
| Attitude towards exercise | Exercise |
| Work schedule | Work-life balance |
| Screen time | Self-care and lifestyle habits |
| Blood pressure | Physical health |
| Metabolic health | Physical health |
| Amount of sleep | Sleep and recovery |
| Quality of sleep | Sleep and recovery |
| Snoring | Sleep and recovery |
