# Supplementary material for: Using online wellness assessment to screen for risk of lowered work ability, burnout, depression and anxiety in occupational health: A cross-sectional study
Source: Digit Health. 2024 Sep 9;10:20552076241274018. doi: 10.1177/20552076241274018 (PMC11384527; doi:10.1177/20552076241274018)

# Supplement 3

Increase in adjusted R^2^ for each occupational health indicator when questions are added to the model. BBI-15 = Bergen Burnout Indicator, GAD-2 = Generalized Anxiety Disorder Scale, WAI = Work Ability Index, WAS = Work Ability Score.


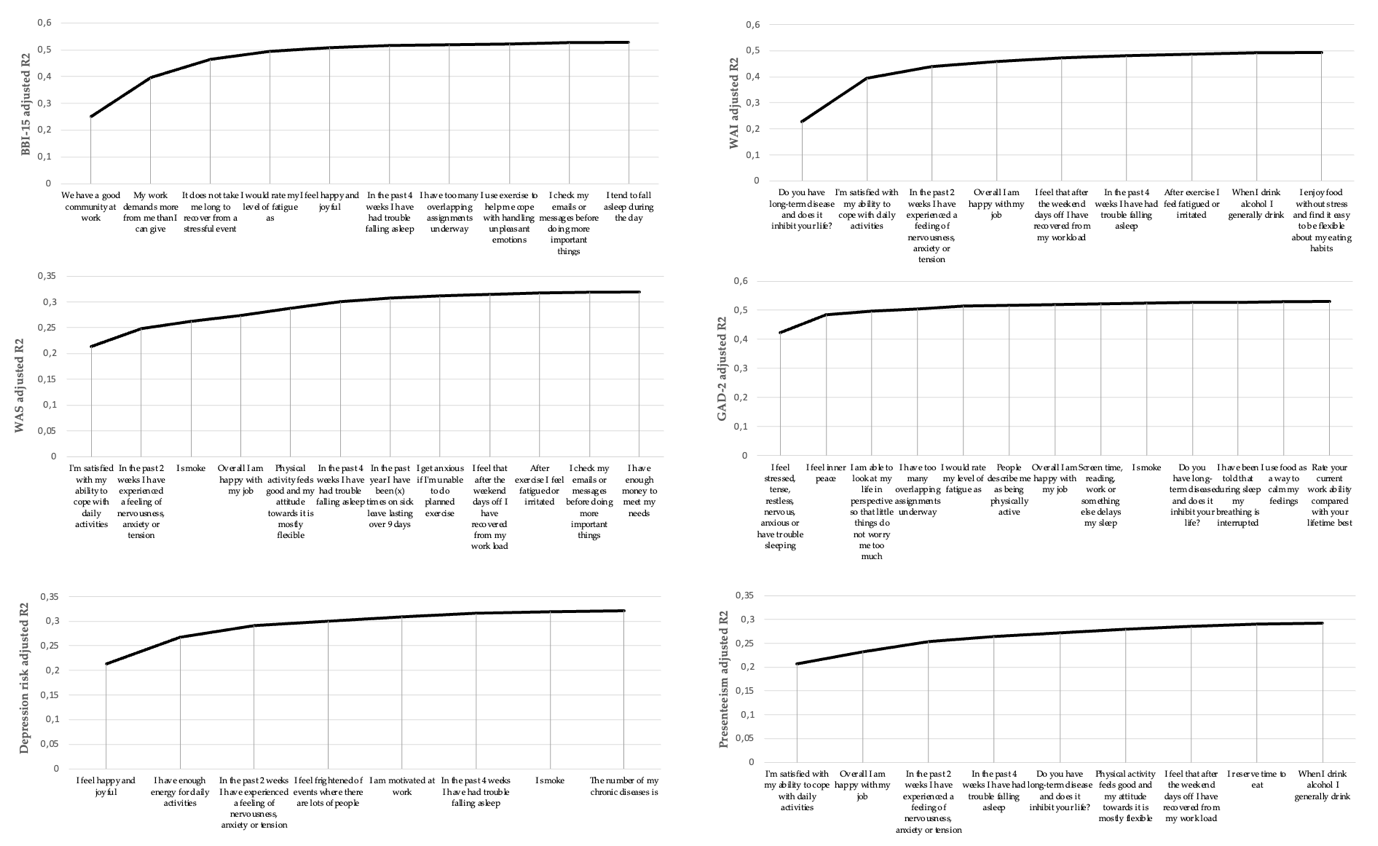

Supplement: sj-docx-3-dhj-10.1177_20552076241274018 - Supplemental material for Using online wellness assessment to screen for risk of lowered work ability, burnout, depression and anxiety in occupational health: A cross-sectional study [file sj-docx-3-dhj-10.1177_20552076241274018.docx]
