# Supplementary material for: Using online wellness assessment to screen for risk of lowered work ability, burnout, depression and anxiety in occupational health: A cross-sectional study
Source: Digit Health. 2024 Sep 9;10:20552076241274018. doi: 10.1177/20552076241274018 (PMC11384527; doi:10.1177/20552076241274018)
Supplement: sj-docx-4-dhj-10.1177_20552076241274018 - Supplemental material for Using online wellness assessment to screen for risk of lowered work ability, burnout, depression and anxiety in occupational health: A cross-sectional study [file sj-docx-4-dhj-10.1177_20552076241274018.docx]

# Supplement 4

Minimum set of questions and their scales.

| Question | 0–20 | 21–40 | 41–60 | 61–80 | 81–100 |
| --- | --- | --- | --- | --- | --- |
| We have a good community at work | Fully disagree | Partly disagree | Neither agree nor disagree | Partly agree | Fully agree |
| I would rate my level of fatigue as | Worst possible fatigue | Very fatigued | Fatigue | Slightly fatigued | No fatigue |
| I’m satisfied with my ability to cope with daily activities | Very unsatisfied | Unsatisfied | Neither agree nor disagree | Satisfied | Very satisfied |
| In the past 2 weeks, I have experienced a feeling of nervousness, anxiety or tension | Every day | Most days | Some days | Once | Never |
| I feel stressed, tense, restless, nervous, anxious or have trouble sleeping | Very much | A lot | Somewhat | Slightly fatigued | Not at all |
| I feel inner peace | Not at all | Slightly | Somewhat | Mostly | Completely |
| I feel happy and joyful | Fully disagree | Partly disagree | Neither agree nor disagree | Partly agree | Fully agree |
| I have enough energy for daily activities | Not at all | Little | Some | Almost enough | Enough |
